# Efficacy, predictability and safety of small incision lenticule extraction (SMILE)

**DOI:** 10.1186/s40662-015-0024-4

**Published:** 2015-08-31

**Authors:** Ekktet Chansue, Morakot Tanehsakdi, Sukanda Swasdibutra, Colm McAlinden

**Affiliations:** TRSC International LASIK Center, 6th Floor U Chu Liang Building, 968 Rama 4 Road, Bangkok, Thailand; Flinders University, Bedford Park, Adelaide, South Australia Australia; Wenzhou Medical College, Wenzhou, Zhejiang China

**Keywords:** Small incision lenticule extraction, VisuMax, Femtosecond

## Abstract

**Background:**

The purpose of this case series is to report the one-year outcomes of small incision lenticule extraction (SMILE) using the VisuMax® femtosecond laser.

**Methods:**

Two hundred and six patients were recruited for this retrospective, single center study at TRSC International LASIK Center in Bangkok, Thailand. Patients underwent SMILE, whereby an intrastromal lenticule was cut using a femtosecond laser and then manually extracted without the need for flap creation. Outcome measures included refraction, visual acuity and contrast sensitivity evaluation. Patients were treated and followed for one year.

**Results:**

SMILE for the correction of low to high myopia was performed on 347 eyes of 206 patients. The mean preoperative spherical equivalent was −4.96 ± 1.88 diopters (D). On the first day following surgery, for eyes with a plano target refraction (99.14 % of all eyes), uncorrected distance visual acuity (UDVA) was 20/20 or better in 90 % of eyes. At the one week postoperative exam, the mean spherical equivalent was 0.01 ± 0.36 D and UDVA was 20/20 or better in 84 % of eyes. After one year follow-up, no eyes showed loss of 2 or more lines of visual acuity and 31 % of eyes gained one or more lines. The photopic contrast sensitivity of SMILE treated eyes at 12 and 18 cycles per degree (cpd) improved from 1.59 and 0.94 preoperatively to 1.6 and 0.98, respectively, after one year.

**Conclusions:**

In this series, SMILE using the VisuMax® femtosecond laser demonstrated that after one year it is an effective, predictable and safe minimally invasive corneal refractive procedure.

## Background

Laser refractive surgery has been performed for decades, and there have been tremendous advancements in terms of technique and technology, making it increasingly precise and highly predictable [1]. Laser in-situ keratomileusis (LASIK) is currently the most common laser refractive procedure for the treatment of myopia – its advantages include early postoperative improvement in visual acuity and minimal postoperative patient discomfort. Although LASIK patients report 95 % satisfaction, a spectrum of complicated side effects can negatively impact results [2]. Patient complaints may include visual distortions such as glare, halos, dry eyes and decreased visual acuity in low light [3]. Serious complications such as infection and inflammation associated with the creation of the corneal flap have further significant consequences for LASIK patients. The limitations of this procedure have already been proven in long term (6-and 10-year follow-up) studies related to the induction of aberrations and regression of correction [4, 5]. The femtosecond laser has been used to cut LASIK corneal flaps with high precision for over a decade [6, 7]. The ability to perform these highly accurate cuts to the corneal tissue has sparked new enquiry into a method of lamellar refractive surgery that may be less invasive.

Small incision lenticule extraction (SMILE) offers a paradigm shift in laser vision correction by using a less invasive technique that creates a lenticule inside the intact cornea and subsequently removing it through an incision, typically less than 4 mm in size [8]. ReLEx® SMILE is performed using the VisuMax® femtosecond laser (Carl Zeiss Meditec AG, Jena, Germany). Instead of creating a corneal flap, a small side-cut incision is created in the cornea for lenticule extraction. Theoretically, by leaving the anterior cornea intact, surgeons are able to maintain its biomechanical stability while better protecting the nerve fibers. The development of a procedure that limits the damage to corneal nerve fibers and preserves the biomechanical strength of the cornea reduces the potential for patient discomfort and flap-induced complications including incomplete and irregular flap cuts, thin flaps, buttonholes and free caps, as well as the associated risk of induced astigmatism, and dry eye. Very promising initial safety and efficacy results were first published by Sekundo et al. in 2008, utilizing femtosecond lasers to create intrastromal lenticule cut patterns to facilitate refractive lenticule extraction through small incisions, eliminating the need for flap creation [9]. Further studies have confirmed that SMILE appears to be safe, predictable and efficacious in the correction of myopia [10–13].

The purpose of this study was to evaluate up to one year results of 347 eyes treated for myopia and myopic astigmatism using ReLEx® SMILE procedure with the VisuMax® femtosecond laser. This method includes the creation and extraction of a refractive lenticule through a small incision as an alternative to the creation and lifting of a hinged flap.

## Methods

All participants were recruited at TRSC International LASIK Center in Bangkok, Thailand, and were provided written informed consent that explained the details of the procedure and study protocol in accordance with the principles of the Declaration of Helsinki. In order to be included, patients had to be a minimum of 18 years of age, have a myopic correction which had been stable for a year or longer, best corrected visual acuity of 20/50 or better and the ability to attend postoperative assessment time points at 1 day, 1 week, 1 month, 3 months, 6 months and 1 year. Also, patients included had a minimum corneal thickness of 475 μm, and minimum residual stromal bed of 275 μm. Patients were excluded if they had any ocular conditions other than myopia and/or astigmatism. All surgeries were conducted by EC.

### Assessments/outcome measurements

In order to assess the patient’s eligibility to participate in the study procedure, all patients underwent a complete eye examination, which included objective and manifest visual acuity and refractions, pupil size evaluation, intraocular pressure measurement, keratometric measurement, slitlamp examination, complete fundoscopic evaluation and contrast sensitivity assessment (Vistech Contrast Sensitivity Chart). At each postoperative appointment, patients were assessed for best corrected distance visual acuity (CDVA), uncorrected distance visual acuity (UDVA) (Both were measured using the ETDRS Visual Acuity Chart), objective and manifest refractions, slitlamp examination, contrast sensitivity assessment and applanation tonometry.

### Surgical technique

All surgeries in this study were performed by EC. After application of topical anesthesia (Tetracaine Hydrochloride 0.5 %, Alcon Corporation, Switzerland), standard sterile draping and insertion of the speculum, the patient’s eye was centered and docked with the curved interface cone before application of suction fixation. Unlike excimer laser techniques, ReLEx® SMILE clearly defines the area where cuts will be performed. The laser, for photo-dissection, is activated and initially cuts the posterior surface of the refractive lenticule (spiral-in shot pattern) followed by creation of the lenticule border. The anterior surface of the refractive lenticule (spiral-out) is then formed which extends beyond the posterior lenticule diameter by 0.5 mm to form the anterior stromal layer (ASL), and is followed by a vertical curvilinear cut to form the entrance wound. We used the following femtosecond laser parameters: 100 μm ASL thickness, 7.5 mm anterior-plane cut diameter, 6.5 mm optical zone of lenticule, 160 nJ of energy with lenticule side-cut angles at 135°. A 2.1 mm entrance wound was created centered between 9 and 12 o’clock in all cases. The spot distance and tracking spacing are 4.5/4.5 μm for the posterior lenticule plane, 2.5/2.5 μm for the lenticule side-cut, 4.5/4.5 μm for the anterior lenticule plane and 2.5/2.5 μm for the entrance wound side-cut. After the suction was released, a Sinsky hook was first used to separate the entrance wound cut made by the femtosecond laser, and then to identify the edge of the lenticule under the ASL. A Chansue ReLEx® Dissector (CRD) was then used to separate the posterior surface of the ASL from the anterior surface of the lenticule and then to release the lenticule from its bed. The lenticule was then grasped and extracted with a pair of non-toothed serrated microforceps through the small incision.

### Statistical analysis

All patient demographic and baseline information, as well as outcome measurement data were entered into Microsoft Excel (Microsoft Corporation, Redmond, Washington, USA). Statistical analyses were performed using the data analysis features of Microsoft Excel. Analysis of visual acuity results were performed by calculating the geometric mean with standard deviation into logMAR format from Snellen examination results [14].

## Results

A total of 347 eyes (206 patients, 188 right eyes and 159 left eyes) were treated for myopia and myopic astigmatism using the ReLEx® SMILE procedure. Baseline characteristics of treated patients are listed in Table 1. Of particular note is the eyes with up to −10.0 D of myopia and −3.75 D of astigmatism included in this series. Three hundred nineteen eyes (92 %) were evaluated at one year postoperatively.

**Table 1:** Patient characteristics at baseline and one year

	Baseline (*n* = 347)	One Year (*n* = 319)
		92 % follow-up rate
Age at time of Surgery (years)		
	31 ±7 (18–56)	
Gender	31 % Male/69 % Female	
Spherical Equivalent (D)		
	−4.96 ± 1.88 (−1.0 to −10.5)	0.09 ± 0.31 (1.125 to −1.25)
Sphere (D)		
	−4.61 ± 1.85 (−0.75 to −10.0)	0.17 ± 0.3 (1.25 to −1.75)
Cylinder (D)		
	−0.71 ± 0.61 (0.0 to −3.75)	−0.17 ± 0.34 (1.0 to −1.25)

**Fig. 1:**
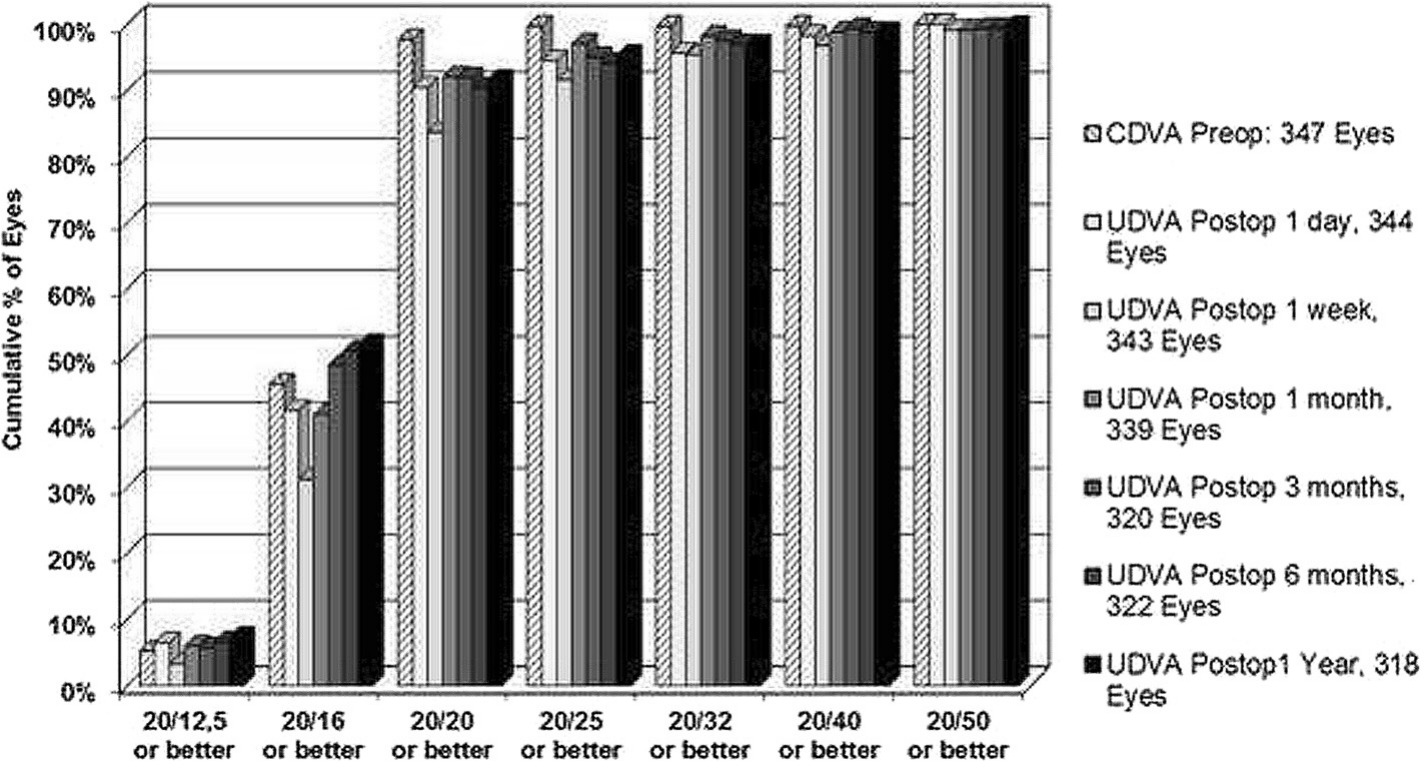
Cumulative proportion of eyes with plano target refraction achieving specific degrees of UDVA at each of the follow-up time points, 1 day, 1 month, 3 months, 6 months and 1 year

All eyes had a CDVA of 20/25 or better preoperatively and 94 % had a UDVA of 20/25 or better at 1 day postoperatively. UDVA remained stable, with 95 % of eyes achieving 20/25 or better after one year. Figure 1 illustrates the percentage of all eyes (*n* = 347) in which the target refraction was plano that reached the desired levels of UDVA. There were no eyes, at any of the specified time points up to one year, that lost two or more lines of CDVA.

**Fig. 2:**
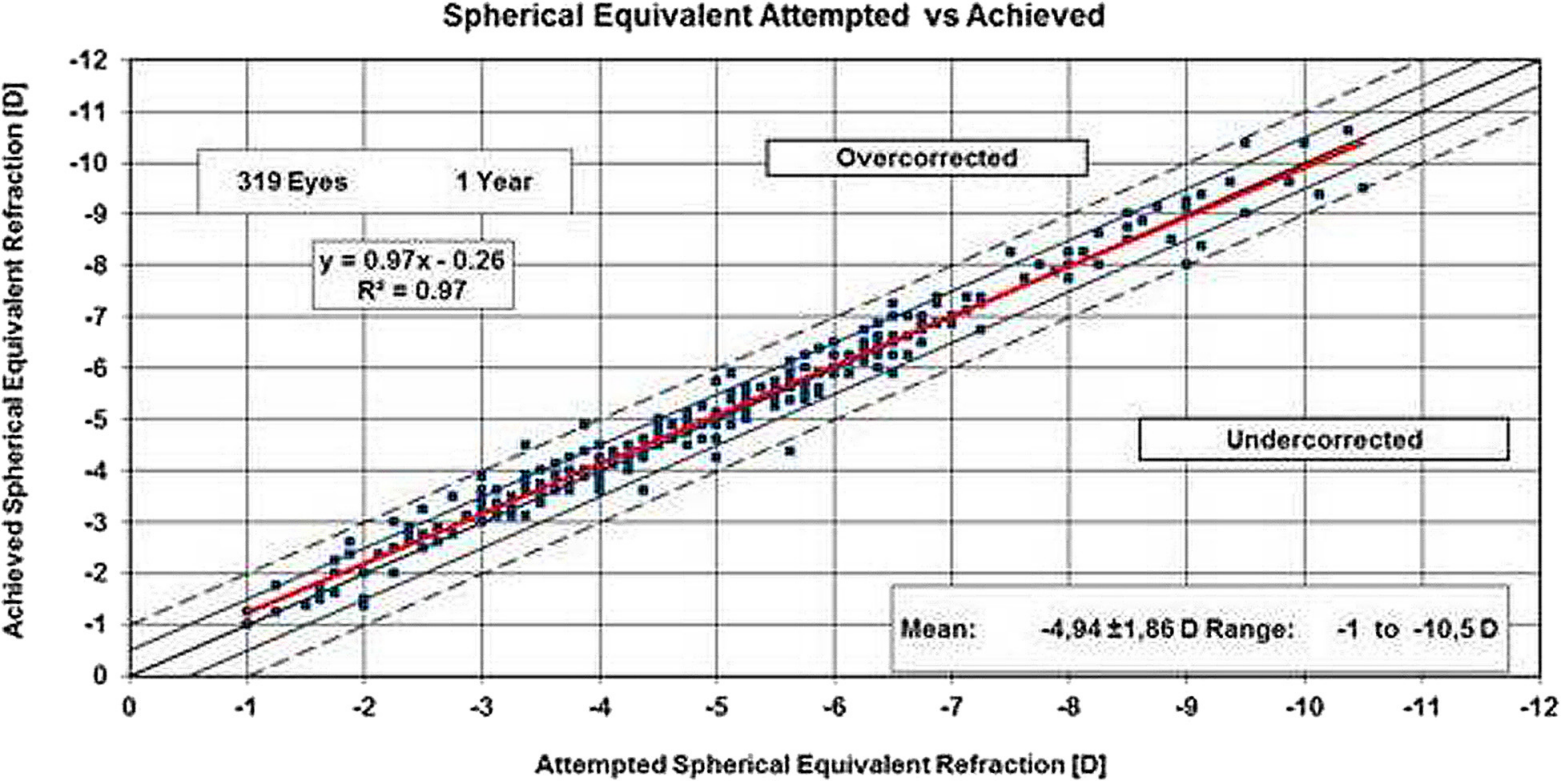
Scatterplot of the attempted spherical equivalent refraction against the achieved refractive change at 1 year

Figure 2 illustrates the spherical equivalent (SE) at one year postoperatively as a scatterplot of attempted versus achieved refraction for all eyes. The mean SE at one year was 0.09 ± 0.31 D. The mean astigmatism at one year (*n* = 319) was 0.17 ± 0.34 D.

**Fig. 3:**
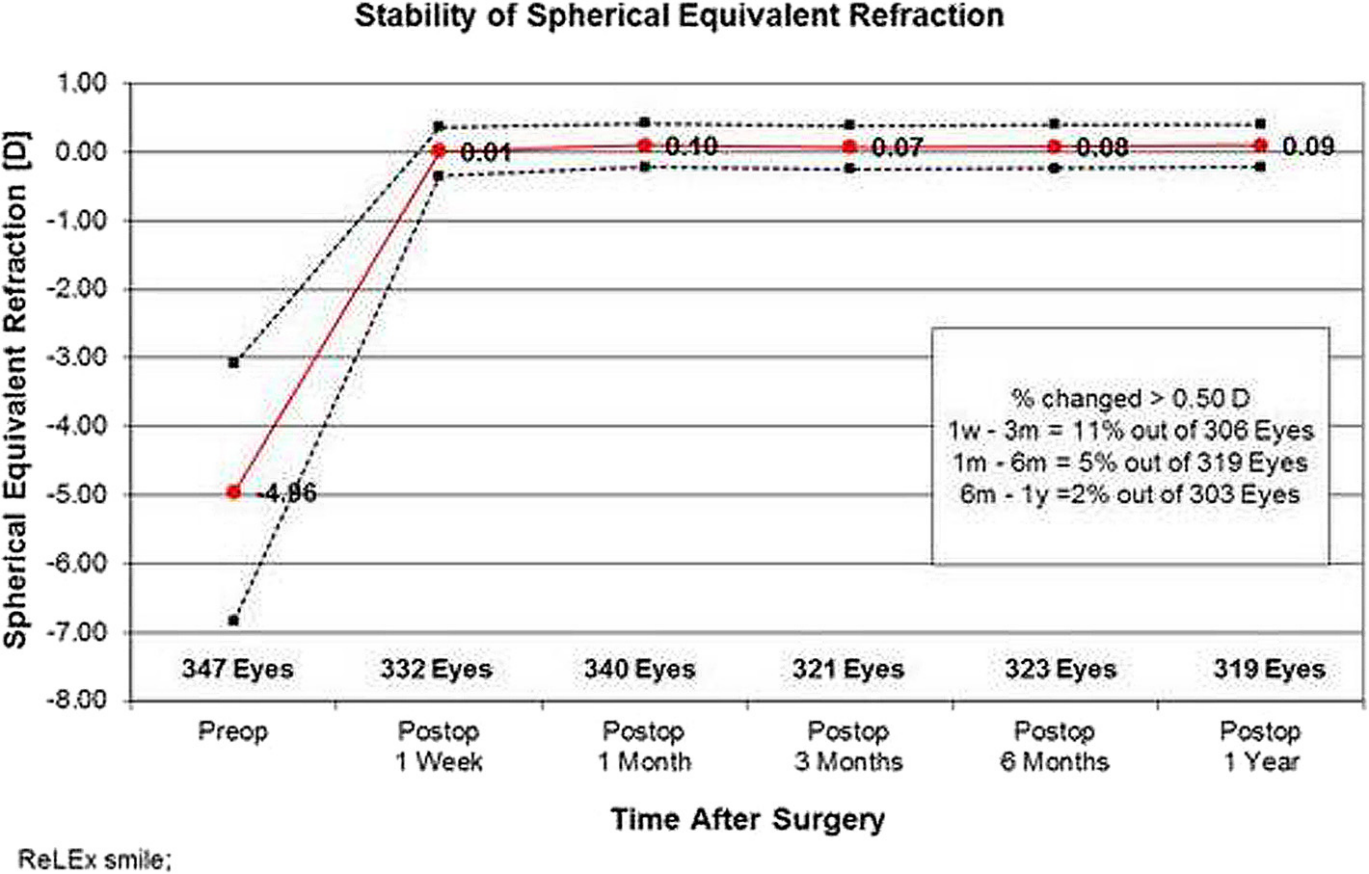
Mean spherical equivalent plotted as a function of time at each of the postoperative time points, illustrating the stability of the refraction

At 1 year, 93 % of ReLEx® SMILE treated eyes were within ± 0.5 D of the intended refractive target and 99 % were within ± 1.0 D. Figure 3 illustrates the stability of the refractive change over time by plotting the mean SE at each of the follow-up time points, 0.10 D at 1 month, 0.07 D at 3 months, 0.08 D at 6 months and 0.09 D after 1 year.

There was a moderate increase in photopic contrast sensitivity (CS) after 1 year at 12 cpd (1.59 to 1.6) and 18 cpd (0.94 to 0.98), and a similar decrease at 3 cpd (1.82 to 1.78) and 6 cpd (1.99 to 1.97) (Table 2).

**Table 2:** Changes in photopic contrast sensitivity preoperatively and after 3 months, 6 months and 1 year

	3 cpd	6 cpd	12 cpd	18 cpd
Preop 329 Eyes	1.82 ± 0.15	1.99 ± 0.14	1.59 ± 0.19	0.94 ± 0.25
3 months 305 Eyes	1.79 ± 0.13	1.97 ± 0.14	1.59 ± 0.16	0.94 ± 0.22
6 months 295 Eyes	1.78 ± 0.12	1.97 ± 0.13	1.6 ± 0.16	0.98 ± 0.21
1 year 281 Eyes	1.78 ± 0.12	1.97 ± 0.12	1.6 ± 0.14	0.98 ± 0.19

Mean ± SD

No significant side effects or complications were observed in any of the eyes in this study.

## Discussion

Good visual outcomes, predictability of refractive correction, stability and safety are integral to the success of refractive surgical procedures and are often the key outcome measures. In this current case series, we have demonstrated that refractive correction with ReLEx® SMILE produced very promising results, adding to the existing clinical evidence by previous SMILE studies by providing follow-up outcomes of up to one year.

The published predictability of LASIK has ranged widely from 78.2 % to 96.7 %, compared to 90.0 % to 95.6 % in previous predictability reports using SMILE [9, 10, 12, 15–17]. In a recent study by Hjortdal et al., they determined that 3 months after patients were treated with SMILE, the achieved refraction was 0.25 ± 0.44 D less than the attempted correction, and that 94 % of patients were within ±1.00 D [18]. Regression analysis determined that undercorrection could be predicted by increasing patient age (0.10 D per decade) and steeper corneal curvature (0.04 D per D) [18]. Shah et al. found in their 2011 study that 91 % of eyes at 6 months were within ±0.5 D of the intended correction, and 98 % were within ±1.00 D when beginning with a mean preoperative SE of −4.87 ± 2.16 D [10]. Similarly, we found that with our mean preoperative SE of −4.96 ± 1.88 D, 93 % were within ±0.5 D of the intended correction and 99 % were within ±1.00 D at 6 months. These values were maintained at the 1 year follow-up time point. Recently, Kamiya et al. reported in their group of 52 eyes that 100 % of eyes were within ±0.5 D of the intended correction after SMILE, suggesting that their predictability results were slightly higher than previous studies due to slightly lower myopic correction and the use of the newer generation femtosecond laser [19].

Unlike the variable environmental factors, which are difficult to mitigate and can cause inconsistencies in all excimer laser ablations, ReLEx® SMILE is performed within a closed system. The precision of the laser cut is independent from potential interference from particles, tissue hydration or fluctuating humidity levels of the ambient air within the surgical suite, potentially eliminating the need for the development of nomograms, tailored to specific locations or surgeons. Additionally, ReLEx® SMILE leaves the anterior lamellae intact. Being the strongest part of the stroma, the anterior lamellae have biomechanical advantages over both photorefractive keratectomy (PRK) and LASIK [20]. Reinstein and colleagues developed a mathematical model that calculated the stromal tensile strength after PRK, LASIK and SMILE. They were able to predict that the postoperative tensile strength after SMILE was approximately 10 % higher than PRK and 25 % higher than LASIK [20]. They further demonstrated that the postoperative stromal tensile strength decreased with increasing flap thickness by 0.22 %/μm in LASIK, but increased by 0.08 %/μm for greater cap thickness in SMILE. The model predicted that SMILE lenticule thickness could be approximately 100 μm greater than the LASIK ablation depth and still have equivalent corneal strength (equivalent to approximately 7.75 D), which means that SMILE can be expected to correct higher levels of myopia within the cornea than is currently possible with LASIK or PRK [20]. In this series, we were able to treat a higher level of myopia (SE up to −10.5 D) and achieve optimum optical quality as well as reduce aberrations, known to negatively affect night vision [21]. In addition to devices which measure aberrations, questionnaires may be used to assess the night vision quality following refractive surgery [22, 23].

Stability of the achieved refractive change is important to monitor and highly influence clinical outcome and patient satisfaction. Previous studies have shown good refractive stability over the follow-up periods. Shah described refractive stability by one week postoperatively and no further significant change at 1 month [10]. Similarly, Sekundo and colleagues reported that the patients in their study had a stable refraction after one week and no further significant change in SE was observed at 1 month (0.05 D), 3 months (0.14 D) and 6 months (0.10 D) [10, 12]. In our study, the changes in SE are in line with previous studies. We were also able to follow our cohort for 1 year, observing a mean SE of 0.10 D at 1 month (340 eyes), 0.07 D at 3 months (321 eyes), 0.08 D at 6 months (323 eyes) and 0.09 D after 1 year (319 eyes). Additionally, in the patients that we have examined after 2 years (214 eyes), the changes to SE were only 0.06 D.

With regards to visual acuity, we found that 94 % of our treated eyes with a target refraction of plano (99.14 % of all eyes), achieved a UDVA of 20/25 or better at 1 day postoperatively and 95 % had this same result after one year. In previous studies, Vestergaard et al. reported that 40 % and 73 % of their patients had UDVA of 0.1 logMAR (20/25) or less postoperatively, at 1 day and 3 months, respectively; Shah et al. found that 79 % of their patients achieved a UDVA of 20/25 or better at 6 months postoperatively [10, 11]. Looking at visual acuity lines gained or lost after one year, 31 % of our treated eyes gained one line, 1 % gained two lines, and 8 % lost one line. Our results were quite similar to the findings in 2011 by Sekundo et al., who after 6 months reported that 32.3 % of the patients gained one line, 3.3 % gained two lines of best corrected spectacle visual acuity, and 8.8 % lost one line [12].

Halos, glare and night vision complaints have been reported by 17-20 % of LASIK patients which may be caused by higher-order aberrations, particularly in low light levels when the pupil is large [24, 25]. It is widely accepted that with higher levels of LASIK correction, there is a greater potential for increases in higher-order aberrations. In addition, ignoring the effects of ablation on higher order aberrations, LASIK flap creation itself increases higher order aberrations [26]. However, the single, small vertical cut used in ReLEx® SMILE minimizes collapse or stromal damage, inducing fewer aberrations, leading to better quality of vision.

## Conclusions

Based on our results in using ReLEx® SMILE for the correction of myopia and myopic astigmatism, we conclude that the procedure is safe, highly predictable and very efficacious. However, in future studies, we recommend a longer follow-up period after surgery.
